# Health risk assessment for particulate matter: application of AirQ+ model in the northern Caribbean region of Colombia

**DOI:** 10.1007/s11869-023-01304-5

**Published:** 2023-02-15

**Authors:** Heli A. Arregocés, Roberto Rojano, Gloria Restrepo

**Affiliations:** 1 Grupo de Investigación GISA, Facultad de Ingeniería, Universidad de La Guajira, Riohacha, Colombia; 2 Grupo Procesos Fisicoquímicos Aplicados, Facultad de Ingeniería, Universidad de Antioquia SIU/UdeA, Calle 70 No. 52–21, Medellín, Colombia

**Keywords:** AirQ+, PM_10_, PM_2.5_, Health issues, Relative risk

## Abstract

Air pollution is considered the world’s most important environmental and public health risk. The annual exposure for particulate matter (PM) in the northern Caribbean region of Colombia between 2011 and 2019 was determined using PM records from 25 monitoring stations located within the area. The impact of exposure to particulate matter was assessed through the updated Global Burden of Disease health risk functions using the AirQ+ model for mortality attributable to acute lower respiratory disease (in children ≤ 4 years); mortality in adults aged > 18 years old attributable to chronic obstructive pulmonary disease, ischaemic heart disease, lung cancer, and stroke; and all-cause post-neonatal infant mortality. The proportions of the prevalence of bronchitis in children and the incidence of chronic bronchitis in adults attributable to PM exposure were also estimated for the population at risk. Weather Research and Forecasting-California PUFF (WRF-CALPUFF) modeling systems were used to estimate the spatiotemporal trends and calculate mortality relative risk due to prolonged PM_2.5_ exposure. Proportions of mortality attributable to long-term exposure to PM_2.5_ were estimated to be around 11.6% of ALRI deaths in children ≤ 4 years of age, 16.1% for COPD, and 26.6% for IHD in adults. For LC and stroke, annual proportions attributable to PM exposure were estimated to be 9.1% and 18.9%, respectively. An estimated 738 deaths per year are directly attributed to particulate matter pollution. The highest number of deaths per year is recorded in the adult population over 18 years old with a mean of 401 events. The mean risk in terms of the prevalence of bronchitis attributable to air pollution in children was determined to be 109 per 100,000 inhabitants per year. The maximum RR values for mortality (up 1.95%) from long-term PM_2.5_ exposure were predicted to correspond to regions downwind to the industrial zone.

**Supplementary information:**

The online version contains supplementary material available at 10.1007/s11869-023-01304-5.

## Introduction

Worldwide, nine out of ten people breathe polluted air every day (Ćurić et al. 2022). The World Health Organization (WHO) estimates that approximately 8.7 million people die annually from exposure to atmospheric pollutants (WHO 2018, 2021a), which makes air pollution one of the greatest environmental threats to human health, along with climate change (Guzmán et al. 2022). The health risks from air pollution associated with particulate matter (PM) smaller than 10 and 2.5 microns (µm) in diameter (PM_10_ and PM_2.5_, respectively) are of particular relevance to public health. Both PM_10_ and PM_2.5_ comprise a complex mixture of solids and aerosols composed of small liquid droplets, dry solid fragments, and solid cores with liquid coatings (Eastwood 2008). They also contain a mixture of chemical species. PM_10_ is inhalable into the lungs and is retained in the extra-thoracic region, while PM_2.5_ has the ability to deposit in the pulmonary alveoli and reach the bloodstream (Martin et al. 2014). Recent research shows that short- and long-term exposure to PM_10_ and PM_2.5_ causes health conditions, particularly in populations of low socioeconomic status (Ouidir et al. 2017), pregnant women (Enders et al. 2019), infants (Torres et al. 2018), and the elderly (Han et al. 2017; Hassanvand et al. 2017). Adverse effects are related to cardiovascular disease, lung cancer, respiratory infections, and aggravation of pre-existing conditions (Carugno et al. 2018; Momtazan et al. 2018). Recent research revealed that severe acute respiratory syndrome coronavirus 2 (the pathogen that causes COVID-19) can be carried by PM (Setti 2020; Setti et al. 2020; Echeverri et al. 2020). Significant associations have been found between PM levels and the number of infections and deaths due to COVID-19 (Mahato et al. 2020; Wu et al. 2020), and air pollution is associated with cancer in humans (Malhotra et al. 2016).

More than 150 million people in Latin America live in cities that exceed the limits set out in the WHO Air Quality Guidelines, and about 6.9% of premature deaths are related to ambient concentrations of PM_2.5_ (Koengkan et al. 2021). In Colombia, the estimated annual health cost attributed to air pollution is USD 30.4 billion, which is equivalent to 1.5% of GDP (MinSalud 2021). From 2004 to 2017, the number of deaths attributable to air pollution in Colombia increased by approximately 49%, according to estimates based on a short analysis period in the main urban centers of the country (INS 2018).

In the northern Caribbean region of Colombia, there are many factors that can affect not only levels of exposure to PM and variability thereof, but also the estimation of the burden of disease attributable to air pollution. This region contains the largest coal complex in Latin America, which covers an area of approximately 69,661 ha. The PM_10_ emissions from mining activities in the area can be carried over long distances although approximately 60% of these emissions can be inhaled within a radius of < 23 km from the source (Arregocés et al. 2018). The annual variability of PM concentrations in northern Colombia is also determined by the arrival of dust from the Sahara. Dust column mass density has been estimated to vary by a factor of 10 (up to 659 kg km^−2^) when such events occur (Méndez et al. 2018). Significant associations have been found between dust storm events and hospital admissions due to respiratory diseases (Kang et al. 2012), as well as between PM concentrations due to desert dust and cardiovascular events and daily deaths (Middleton et al. 2008; Neophytou et al. 2013). Air pollution is a threat to health, especially in low-income populations. Compared to most developed countries that have carried out industrialization programs over several years, low-and middle-income countries have undergone intense urbanization processes that have resulted in dense urban centers with poor air quality (Hystad et al. 2020). In the northern Caribbean region of Colombia, the poverty index is almost double the national value, while the extreme poverty index is three times higher, which may magnify the impact of exposure to air pollutants in terms of public health indices.

The growing burden of disease from air pollution is among the top challenges faced by national governments and public health officials, with far-reaching implications for national economies and human well-being (Health Effects Institute 2019). It is important to quantify the health burden and impacts of air pollution using tools that effectively aid environmental policymakers and authorities in their planning process, and this is required in Colombia to achieve goals in Colombia’s sustainable development plans that seek to reduce the mortality rate attributed to ambient air pollution. Under the implementation of CONPES 3943 of 2018, the “Colombian Policy for the Improvement of Air Quality” (application of Law 1931 of 2018), the Colombian government seeks to reduce the exposure limits of fine particulate matter in urban and industrial centers by 40% (UNIDO 2019).

AirQ+ is a software tool for quantifying the health impacts of air pollution that were developed by the WHO Regional Office for Europe (WHO 2020). AirQ+ allows users to estimate potential effects on human health caused by exposure to air pollution. All calculations performed by AirQ+ are based on methodologies and concentration–response functions from systematic reviews and meta-analysis of epidemiological studies. This software has been used extensively in recent studies. Luo et al. (2020) quantified the health effects of pollutants in six economically important cities in northwest China by means of the AirQ+ model. The authors estimated that PM_10_ had a more significant effect on human health than the other pollutants studied (PM_2.5_, O_2_, NO_2_, and CO), resulting in higher mortality (excess cases of 76.6 and 194.9 for respiratory and cardiovascular mortality, respectively). The AirQ+ model has also been used to estimate the health benefits of reducing PM levels in densely populated cities (Mirzaei et al. 2021). Brito et al. (2022) applied the AirQ+ model combined with a linear mixed model to health indicator data and time series data of pollutant concentrations to evaluate deaths attributable to exposure to mixtures of NO_2_ and PM_2.5_. This approach made it possible to determine the spatial distribution of the proportion of deaths attributable to prolonged exposure to PM in Portuguese municipalities. On the other hand, Rovira et al. (2020) integrated different statistical techniques with burden of disease using disability-adjusted life years and the fraction attributable to the population determined by the AirQ+ model in a region of Spain. The authors estimated that a reduction in fine PM levels to below 10 µg m^−3^ would reduce adult mortality by 0.5–7% in the area.

This study presents PM_10_ concentrations from 25 stations in the northern Caribbean region of Colombia. The purpose of this study was twofold, as follows: first, to evaluate mortality and morbidity due to PM_2.5_ exposure between 2011 and 2019, PM_2.5_ levels were estimated through the PM_10_/PM_2.5_ ratio for each zone within the domain; second, to understand the influence of local and regional sources of PM_10_ particles on the estimates and variability in mortality due to acute lower respiratory disease (ALRI) in children aged 0–4; chronic obstructive pulmonary disease (COPD), ischaemic heart disease (IHD), lung cancer (LC), and stroke in adults aged > 18 years; and post-neonatal infant mortality. A complementary analysis of the risks of prolonged PM_10_ exposure in the study region was carried out using the Lagrange puff model. While fine particulate matter (PM_2.5_) may be more closely associated with adverse respiratory health effects than larger particulate matter (PM_10_) (Osornio-Vargas et al. 2003; Choi et al. 2004), the largest sources of PM pollution emissions in the study region are related to open-pit coal mining, quarrying, unpaved roads, and dust resuspension. These sources discharge coarser fractions of particulate matter into the atmosphere. In addition, some areas only have PM_2.5_ monitoring records available for the years 2018 to 2021.

## Materials and methods

### Study area

**Fig. 1:**
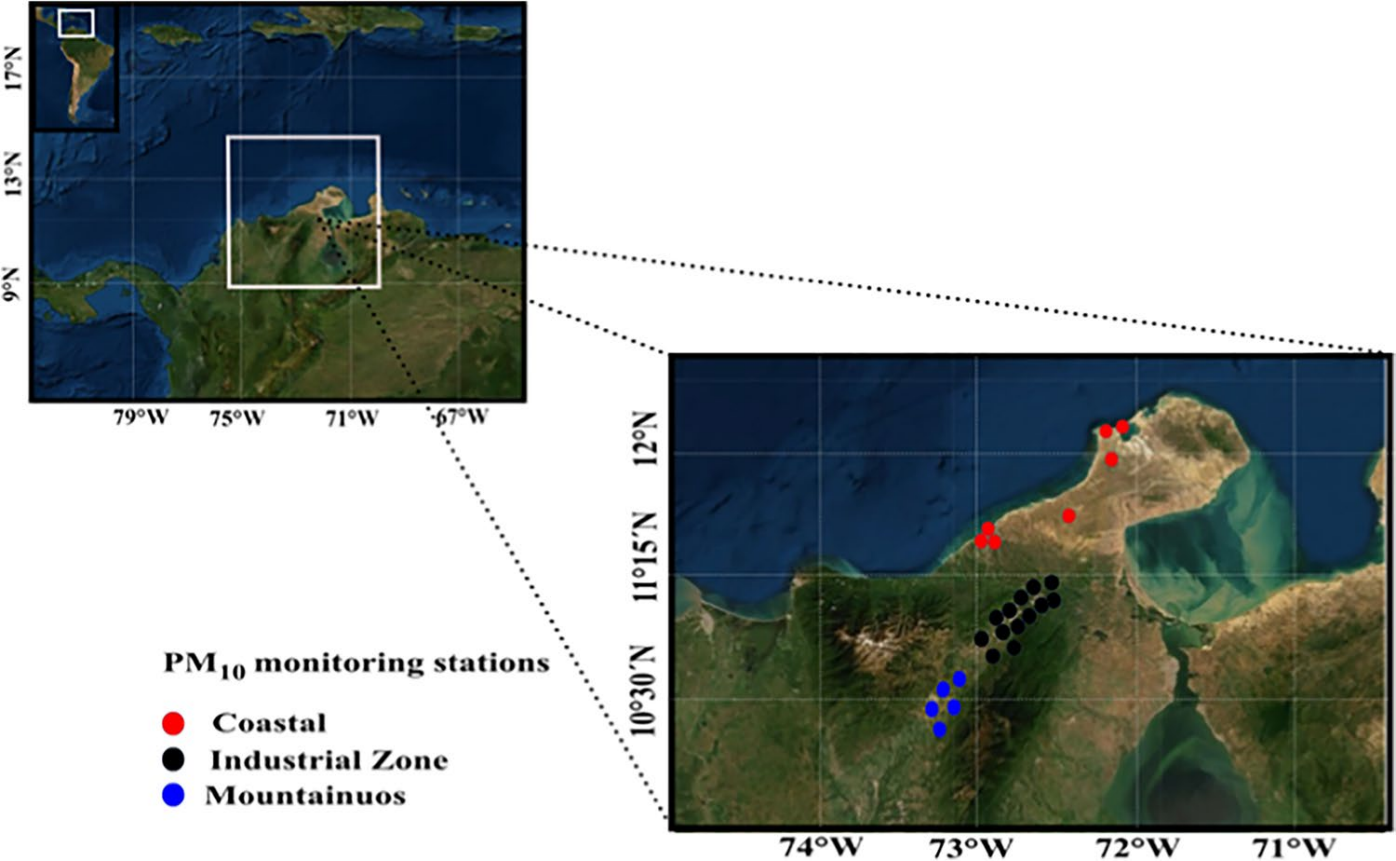
Location of PM_10_ monitoring stations in the northern Caribbean region of Colombia

The northern Caribbean region has a land area of approximately 20,848 km^2^ located at 11°33′N–72°54′W. This area has a complex and flat topography that is characterized by the presence of a mountain range located on the east side, reaching an altitude of 3630 m.a.s.l., and an isolated mountain range in the west, reaching an altitude of 5700 m.a.s.l. According to the last census, the population of the study area is 880,560 (DANE 2018a). Males and females account for 48.8% and 51.2% of the population, respectively. The Northern Caribbean Region consists of 22.6% people ≤ 14 years of age, 68.3% 15–64 year-olds, and 9.1% people > 64 years of age. In the study area, the maximum permissible levels of PM_10_ are 75 µg m^−3^ and 50 µg m^−3^ for 24-h and annual exposure periods, respectively (Fig. 1).

### PM data

Daily 24-h mean PM_10_ concentration data were obtained from the air quality monitoring system (SISAIRE) for 25 stations from January 2011 to December 2019. The monitoring frequency was 1 working day for every 3 days. Valid daily concentrations were accepted for sampling times greater than 23 ± 1 h. For valid annual PM concentrations at each station, a minimum of at least 75% of the data was considered, i.e., 92 samples per year. The following criteria for rejecting the values of concentrations in doubtful samples were applied: (i) sudden occurrence of rare significant sources of PM emissions near the sampling station (e.g., proximal forest fire), which occurred eight times during the study period, especially at some monitoring stations located in mountainous zones and (ii) lengthy power outage (more than 2 h) during the 24-h average sample collection. PM_2.5_ levels were estimated through a conversion factor between PM_2.5_ and PM_10_ from previous studies in the study area (Rojano et al. 2013; Espitia-Pérez et al. 2018). The PM_2.5_/PM_10_ ratio can be used to assess the historical PM_2.5_ pollution in the absence of direct measurements (Xu et al. 2017). Subsequently, PM_2.5_/PM_10_ monthly ratios were estimated using data from PM_2.5_ monitoring records from July 2018 to December 2019 for the three regions (Fig. S1). Monthly PM_2.5_/PM_10_ values were statistically weighted with values from previous studies to establish estimates from 2011 to 2019. The PM_2.5_/PM_10_ ratio for the industrial zone was 0.5 ± 0.3, while for the coastal and mountainous zones, it was 0.4 ± 0.2 (Fig. S1).

Based on the records from the surface stations, PM_10_ estimates were made using a dispersion model (described in more detail in the “AirQ+ ” section), and for the population density of each zone, the representativeness of the exposure of the population at risk was estimated. It was estimated that the PM monitoring stations in the coastal zone can account for 58.1% of the exposure for population groups of inhabitants in these areas. The representativeness of exposure in the population at risk for the stations in the industrial zone was estimated as 69.3%, and the value for stations in the mountain zone was 63.8% (Fig. S2). Stations were classified into three groups based on their location: coastal zone (seven stations), industrial zone (13 stations), and mountainous zone (five stations). These categories were assigned based on the economic activities and physiographic conditions of each region. The coastal zone includes the largest urban centers, is characterized by flat relief mostly covered by desert soils with sparse vegetation, and is where the majority of the indigenous population in the area is concentrated. It has a large number of unpaved roads and areas exposed to wind. It is common for the inhabitants of these rural areas to use charcoal for cooking. The industrial zone is located in the central area of the study domain. This zone contains the largest open-pit coal mine in South America. The climate is humid, with rainfall occurring during the months of March–May and September–November. Conventional open-pit mining methods of drilling and blasting are used for coal extraction with a hydraulic backhoe shovel, and transport trucks of 240 and 320 tonnes are employed. The mountainous zone corresponds to urban settlements located on complex topographic formations. It is a humid zone with arable land that hosts a great diversity of climate types. It is common practice for the rural inhabitants of this area to use charcoal for cooking food.

To estimate the impact of fine dust on health indicators in the Colombian Caribbean region, average fine-dust PM_2.5_ estimates were obtained for the months of Saharan dust arrival (GMAO 2015). Modeled fine-dust PM_2.5_ concentrations on the Earth’s surface, two-dimensional time-averaged monthly diurnal mean, single-level, assimilation, and diagnostic aerosol (M2TUNXAER) output were obtained from the Modern-Era Retrospective analysis for Research and Applications version 2 (MERRA-2) database at a spatial resolution of 0.5° × 0.625° between 2011 and 2019. The MERRA-2 atmospheric reanalysis product was released by the NASA Global Modeling and Assimilation Office (GMAO) in 2017. MERRA-2 obtains aerosol composition measurements by first assimilating satellite aerosol optical depth (AOD) data into the Goddard Earth Observing System Model, version 5 (GEOS-5), from sources such as the Moderate Resolution Imaging Spectroradiometer (MODIS) and by simulating their transport using model winds. It then uses the integrated atmospheric chemistry model of Goddard Chemistry Aerosol Radiation and Transport (GOCART) (Chin et al. 2002; Randles et al. 2017). A descriptive analysis of the monthly average values in the coastal zone, industrial zone, and mountainous zone was performed for the phases before and during the arrival of the Saharan dust to the Colombian Caribbean.

### AirQ+ 

Health issues due to PM_2.5_ and PM_10_ exposure were quantified using the AirQ+ model. AirQ+ allows the integration of health variables directly into air quality management, as well as the definition of public policies related to the control of atmospheric emissions. AirQ+ calculates the attributable proportion of cases, the number of attributable cases per 100,000 people in the population at risk, and the proportion of cases per air pollutant concentration category, based on reference rates from health research, a cutoff value, and values of relative risks.

To quantify the long- and short-term effects of exposure to PM_10_, the following data were provided: (i) annual averages PM between the years 2011 and 2019; (ii) a cutoff value of annual PM concentrations of 15 µg m^−3^ (PM_10_) and 5 µg m^−3^ (PM_2.5_) as recommended by the WHO (WHO 2021b); (iii) data regarding the population at risk, such as the total number of children ≤ 4 years old and adults ≥ 18 years old; and (iv) mortality and mobility data from public health records for the study area. For the dose–response functions set for PM_2.5_, PM_2.5_ levels were estimated using the PM_2.5_/PM_10_ ratio, in accordance with previous studies (Rojano et al. 2013; Espitia-Pérez et al. 2018). AirQ+ quantifies the impact of PM exposure using an attributable proportion (AP) function, defined in Eq. 1. AP is defined as the fraction of the health impacts in a defined population attributable to exposure to an air pollutant, assuming a demonstrated causal relationship between exposure and the health issue and with no significant confounding effects on this association.

1 $$AP=\frac{\sum (RR-1)\times P}{\sum RR\times P}$$

where RR is the relative risk for the health endpoint in a determined exposure to air polluted by PM and P is the fraction of the population under exposure. Table 1 shows the RR values used in this study. The RR values were obtained from systematic reviews, meta-analyses, and research on modifying the variables of long-term PM exposure and all-cause and cause-specific mortality, conducted by Chen and Hoek (2020) and Burnett et al. (2018). These studies show the RRs for concentration-exposure functions and provide suggestions, even at the country level, regarding the type of concentration exposure. The RRs selection criteria were based on an air quality index, socioeconomic status, transport, and energy variability, according to the characteristics of the study area. The number of health issues attributable to PM_10_ exposure (IE) and the number of cases attributable to exposure (NE) can be estimated using Eqs. 2 and 3, respectively.

2 $$IE=I\times AP$$

3 $$NE=IE\times N$$

where *I* is the baseline incidence rate and *N* is the total population exposed to the pollutant. The default relative RR risk values of the AirQ+ model were used for the estimate of hospital admissions for respiratory diseases in this study. These values are obtained from meta-analysis studies (Mudu et al. 2018). The WHO default data available through the AirQ+ software were used since no time series analysis was available for Colombia.

**Table 1:** Relative risks (RR) and baseline incidences with 95% CI implemented in AirQ+ software for the health endpoint estimates at coastal, industrial, and mountainous zones in the northern Caribbean region of Colombia

Outcome	Health endpoint^c^	Baseline incidence^a^			Pollutant	RR (CL: 95%) per 10 μg m^−3b^
		Coastal	Industrial	Mountainous		
Mortality	Respiratory mortality	36.92	20.26	18.29	PM_2.5_	1.0051 (1.0030–1.0073)
	ICD-9-cm 460–519				PM_10_	1.0032 (1.0023–1.0040)
	Cardiovascular mortality	77.47	16.82	27.1	PM_2.5_	1.0044 (1.0033–1.0054)
	ICD-9-cm 390–459				PM_10_	1.0043 (1.0037–1.0049)
	Lung cancer mortality	4.67	4.9	12.19	PM_2.5_	1.11 (1.05–1.18)
	ICD-9-cm 162					
Morbidity	Hospital admissions respiratory disease	768	1421	1007	PM_10_	1.008 (1.0048–1.0112)

^a^Crude rate per 100,000 inhabitants

^b^The values in parentheses represent the low and high relative risks

^c^International Classification of Diseases, 9th Revision, Clinical Modification (ICD-9-cm)

Data on mortality due to ALRI in children aged 0–4; adult mortality (aged > 18 years old) due to COPD, IHD, LC, and stroke; the prevalence of bronchitis in children (aged 0–4); and the incidence of chronic bronchitis in adults (> 18 years old) were obtained from the National Institute of Health of Colombia and DANE (DANE 2018b; INS 2021). AirQ+ utilizes default RRs for population groups based on epidemiological studies conducted in specific age groups. For example, for COPD, IHD, stroke, and LC, it considers the population aged 25 + . However, for our research, we assumed that estimates in the adult population at risk comprise people > 18 years. This assumption was taken because mortality data provided by the government do not include classifications of mortality records by age range that would allow the same AirQ+ estimates to be calculated. To reduce uncertainty, we weighted the RRs used by AirQ+ , adjusted to the population, using results from previous studies in which mortality events were determined for the general population. (Götschi et al. 2008; Cao et al. 2011; Hansell et al. 2016; Kim et al. 2017; Chen and Hoek 2020).

A complementary analysis of the risks of prolonged PM exposure in the study region was performed using the CALPUFF-CALMET-WRF modeling system. Meteorological data from 3 surface stations were input into the CALMET model to generate the meteorological diagnostic fields. Land features were incorporated using USGS global 30 arc-sec SRTM30 data (∼1 km). Land use and land cover data were downloaded from the USGS (https://www.usgs.gov/). Gridded geophysical data, including land use and height, were processed to generate cells for CALMET with 4 km resolution. In the vertical dimension, the 10 vertical layers incorporated into the CALMET modeling had heights of 20, 50, 100, 200, 300, 500, 1000, 2000, 3000, and 50,000 m. We used the Weather Research and Forecasting Model (WRF, National Center for Atmospheric Research) to provide dynamic weather fields for the CALMET model in the CALPUFF model system. The WRF model parameterization is detailed in Supplementary Table S1.

Point and area emissions pertaining to mining activities, commercial activities, biomass burning, and erosion of exposed areas were quantified using USEPA emission factors (Compilation of Emission Factors AP-42). The dispersion model results were compared to measurements from monitoring stations in each zone of the study area in order to validate the results. Performance values of the CALPUFF model are detailed in Supplementary Table S2 and Fig. S3. By using records of PM_10_ concentrations for each zone and daily records of all-cause (natural) mortality for 2011–2019, we evaluated a generalized additive linear model. The model assumed a quasi-Poisson distribution after a logarithmic transformation of the mortality variable (Yang et al. 2020). Based on the Akaike information criterion, non-natural parameter splines were tested to remove model residuals and temporal influence (e.g., rainy season) using Barlett’s test (Priestley 1981). Results were expressed as an increase in the mortality RR per interquartile increase in PM10. Results for each zone were pooled using a random effects model (Lindstrom and Bates 1990). Statistical significance was defined as the lower 95% confidence intervals (CIs) for RR values greater than one. The RR provides the relative rate of risk for health effects of air pollution related to changes in exposure to air pollutants.

## Results and discussion

### PM concentrations

**Fig. 2:**
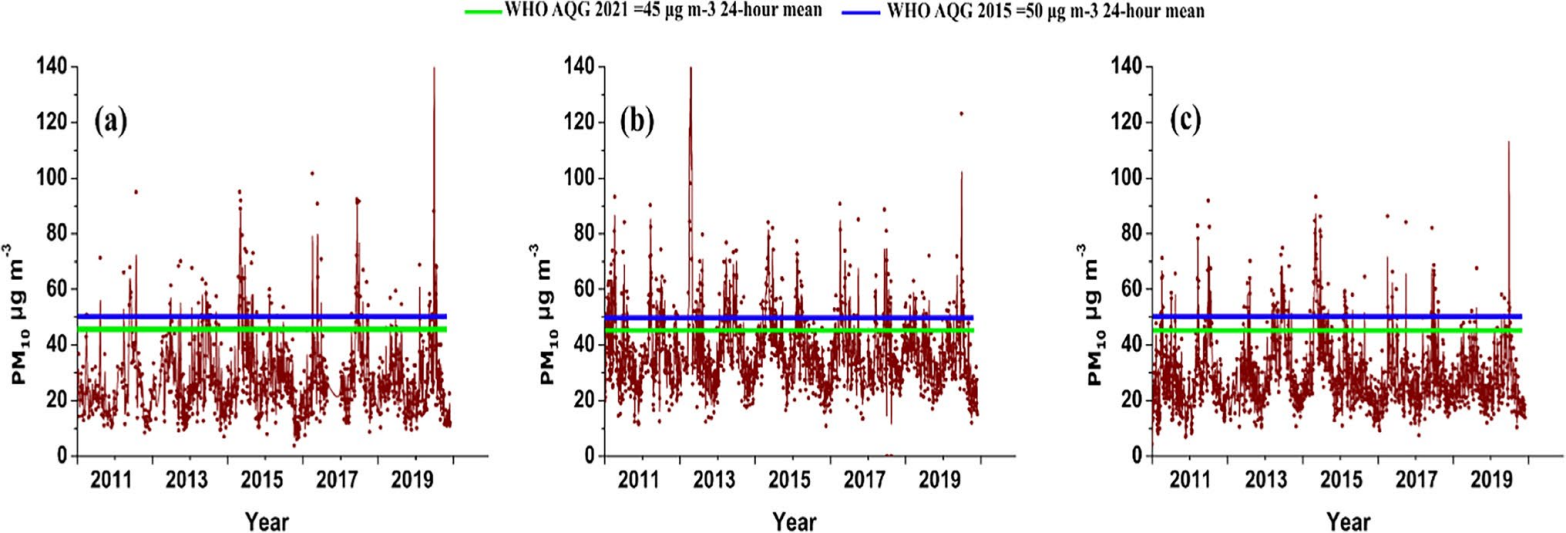
Daily PM_10_ concentrations in the northern Caribbean region of Colombia between 2011 and 2019; **a** stations located in the coastal zone, **b** stations located in the Industrial zone, and **c** stations located in the mountainous zone

The daily mean PM_10_ levels from 2011 to 2019 were 28 µg m^−3^, 38 µg m^−3^, and 30 µg m^−3^ for the group of stations located in the coastal, industrial, and mountainous zones of the northern Caribbean region of Colombia, respectively (Fig. 2). In the coastal zone, 13% of the daily records exceeded the reference value for PM_10_ set by the WHO (45 µg m^−3^), while the reference value was exceeded by 25% and 21% at the stations located in the industrial and mountainous zones, respectively. The highest monthly average PM_10_ across all the stations between February and July was between 26 µg m^−3^ and 50 µg m^−3^. The highest monthly records (42–50 µg m^−3^) were recorded at the stations located in the industrial zone. The lowest monthly values reached averages as low as 16 µg m^−3^ and were recorded in the month of November (Supplementary Table S3).

The highest annual average PM_10_ concentrations were recorded in the coastal (33 µg m^−3^) and industrial zones (44 µg m^−3^) in 2015, while the stations in the mountainous zone reached the highest annual average value (57 µg m^−3^) during 2016 (Table 2). In contrast, the annual minima were recorded at the majority of stations (11 stations) during 2011, with values not exceeding 19 µg m^−3^. In the coastal zone, PM_10_ increased significantly (*p* < 0.05) between the years 2011 and 2015, with levels increasing from 22 to 33 µg m^−3^, and the levels subsequently decreased to 26 µg m^−3^ in 2019. A significant increase in annual PM_10_ concentrations was also observed in the industrial zone between 2011 and 2015, followed by a decrease. During 2021, the WHO established a new reference value of 15 µg m^−3^ for the annual mean safe threshold for PM_10_, based on new evidence. This level was exceeded at every station between 2011 and 2019 (WHO 2021b).

**Table 2:** Annual average concentrations (µg m^−3^) and standard deviation of PM_10_ at coastal, industrial, and mountainous stations in the northern Caribbean region of Colombia between 2011 and 2019

	Coastal zone		Industrial zone		Mountainous zone	
	Mean	StD	Mean	StD	Mean	StD
2011	22	10	37	15	27	15
2012	28	17	39	14	29	14
2013	26	14	43	30	29	15
2014	30	14	39	13	38	15
2015	33	18	44	14	36	13
2016	26	12	38	13	57	14
2017	28	16	39	14	35	19
2018	32	17	37	12	35	14
2019	26	10	36	10	33	14

**Fig. 3:**
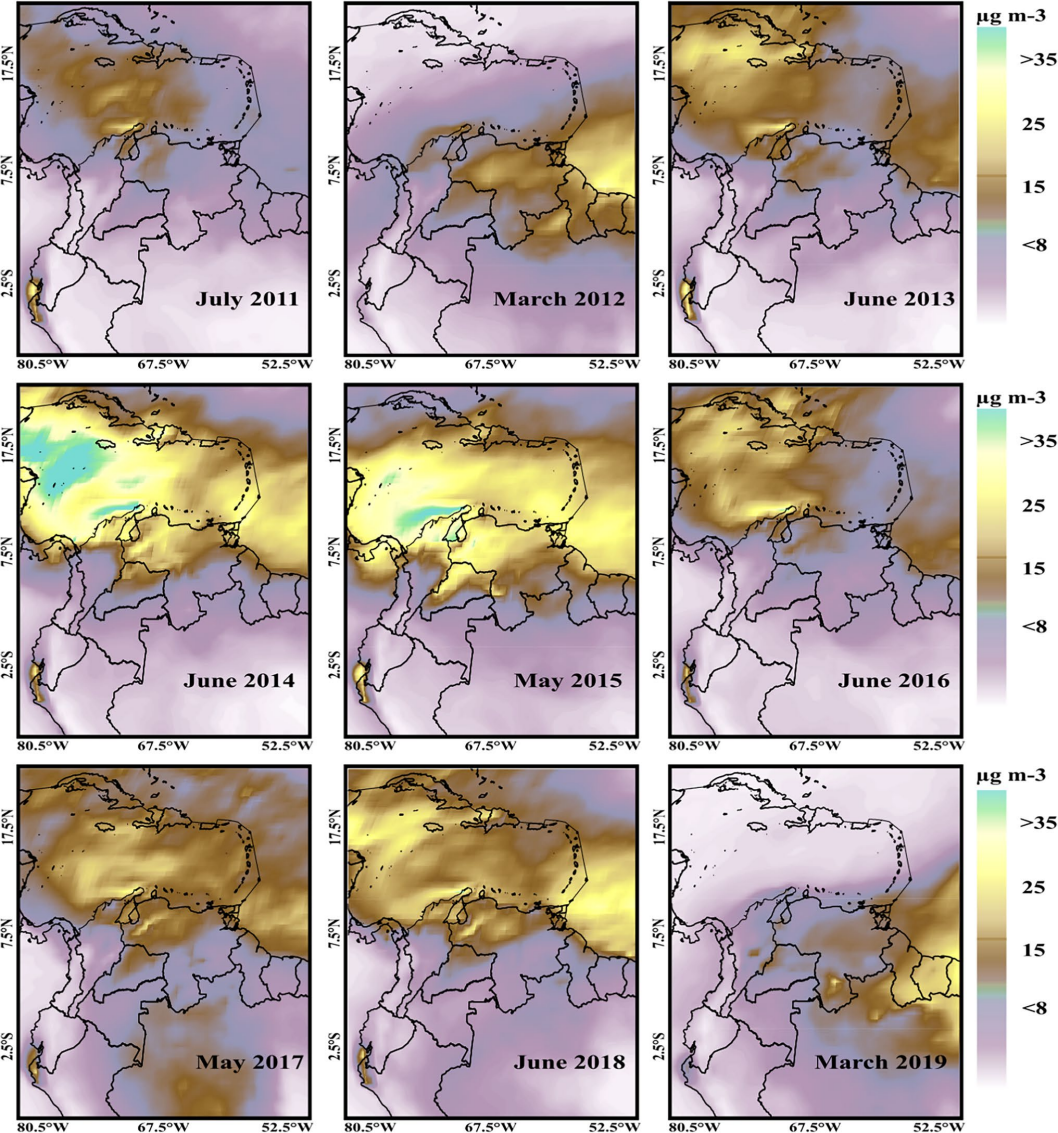
Monthly averages of dust-PM_2.5_ due to Saharan dust being transported to the Colombian Caribbean, for months when episodes of dust occurred, calculated via MERRA-2 reanalysis satellite imagery

Saharan dust, blown thousands of kilometers from its source by wind, represents a major significant natural contributor to atmospheric particulate matter in the northern Colombian Caribbean. Dust associated with PM_2.5_ arriving from the Sahara was spread throughout the Colombian Caribbean (Fig. 3). Based on 9 years of MERRA-2 aerosol reanalysis, we found that PM_2.5_ dust arriving from the Sahara Desert was associated with an average annual increase of 3.7%, 2.2%, and 2.3% in the coastal zone, industrial zone, and mountainous zone, respectively. The largest contributions to PM_2.5_ levels by the Sahara Desert were estimated to have occurred between 2014 and 2015. Annual mean PM_2.5_ increases of up 1.4 µg m^−3^ (3.9% increase, with respect to the annual average) and 1.6 µg m^−3^ (4% increase, with respect to the annual average) were estimated to have occurred in 2014 and 2015, respectively. When Saharan dust begins to arrive, an increase in the average ambient levels of PM_2.5_ is observed compared to the months prior to its arrival. Increases in annual average PM_2.5_ levels upon the arrival of the Saharan dust were recorded in the coastal zone (1.1 ± 0.8 µg m^−3^), the industrial zone (0.8 ± 0.5 µg m^−3^), and the mountainous zone (0.7 ± 0.5 µg m^−3^) during the period 2011–2019.

Transatlantic transport of Saharan dust can affect ambient PM_10_ and PM_2.5_ concentrations in parts of the Americas. For example, Bozlaker et al. (2013) used elemental analysis of the chemical composition of PM in the city of Houston and the chemical mass balance receptor model to determine that during a 3-day episode in the Sahara, the total dust contribution increased by 64% for PM_2.5_ and 85% for PM_10_. On the other hand, it has been determined that the coastal regions of Tabasco, Veracruz, and Yucatan (Mexico) presented a higher percentage of increase in PM_10_ (up to 118%) and PM_2.5_ (59%) levels on Saharan dust days than on non-Saharan days (Kutralam-Muniasamy et al. 2021).

The northern Caribbean region of Colombia has experienced a significant increase in the number of vehicles and urbanization over the past few years, which is reflected in the higher levels of pollutant emissions being released into the atmosphere. Additionally, there has been an increase in the open-pit coal extraction industry (Bayona Velásquez 2016; Worldometers 2020). Another factor that has influenced the PM levels in the region is the contribution of transatlantic dust from the Sahara Desert (Méndez et al. 2018). During the study period, PM_10_ levels in the industrial zone were higher than in the other zones, which is likely due to open-pit coal mining in the area. Mining activities are considered to be an important source of PM (Doria-Argumedo and Fagundo-Castillo 2017), which corresponds with the increasing trend of PM between 2011 and 2015. The impact of the 2014 and 2015 Sahara events on the increase in PM in the region was evident, as can be seen in Fig. 3.

### Long-term health issues

The estimated attributable proportion of mortality due to annual exposure to PM_2.5_ > 5 μg m^−3^ in the northern Caribbean region was between 11.7 and 16.9% for mortality in adults older than 18 years caused by COPD, IHD, LC, and stroke (3609 deaths during the entire study); mortality in children aged 0–4 caused by ALRI (298 deaths during the entire study); and all-cause post-neonatal infant mortality (2739 deaths attributable to PM_10_ levels). Long-term exposure to PM_2.5_ in the northern Caribbean region of Colombia (at PM_10_ concentrations of 34 µg m^−3^) is estimated to cause an average of 738 deaths annually via COPD, IHD, LC, stroke, ALRI, and post-neonatal infant mortality. The mortality rate followed an upward trend from 2011 to 2019. The highest and lowest mortality estimated attributable proportion rates were 16.9% and 16.2%, corresponding to 2015 and 2016, respectively (Table 2). These previous values are associated with high PM_2.5_ exposure in the inhabitants due to population increase compared to the other years of study. However, upon analyzing the annual increase in mortality relative to that of the previous year, it is evident that in 2015, the effects were associated with higher annual PM concentrations during the study period. The annual mortality increase from 2014 to 2015 was 28%. The average annual increase in mortality was estimated to be 11% for the entire region during 2011–2019. This represents the premature death of approximately 107 people each year. The most significant effects of annual mortalities due to COPD, LC, IHD, and stroke have been found to be related to particulate matter rather than pollutants such as NO_2_ and O_3_. Rovira et al. (2020) found that PM exposure below 10 µg m^−3^ is associated with a reduced mortality rate.

The highest APs for the coastal and industrial zones occurred in 2015, at 14.3% and 24.6%, respectively. In comparison, for the mountainous zone, the highest percentage of deaths attributable to PM_10_ pollution occurred in 2016, representing 26.8% of the total cases. Around half (43% to 54%) of the total cases attributable to PM exposure between 2011 and 2019 were attributable to IHD in adults aged > 18 years old, while 16% to 22% of the total cases occurred due to stroke (Supplementary Table S4).

**Fig. 4:**
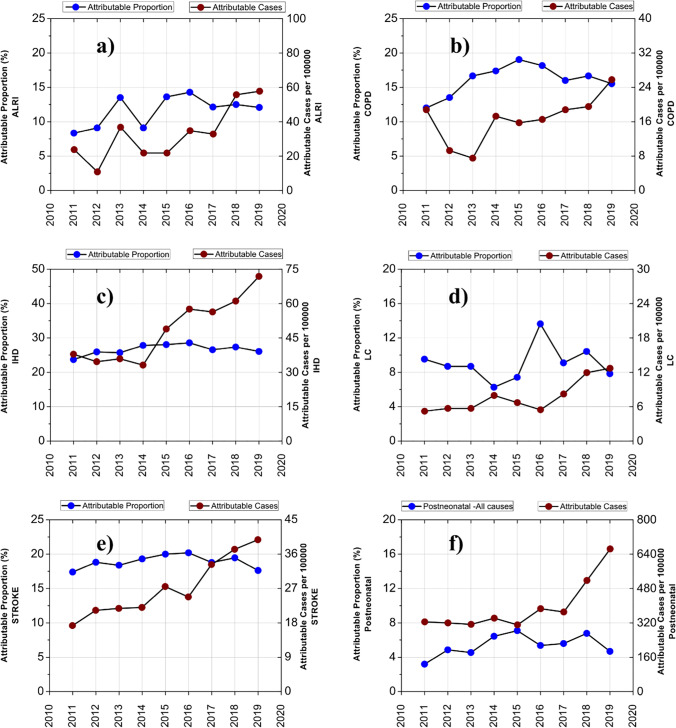
Health impacts attributable to long-term exposure to PM_10_ in the northern Caribbean region of Colombia. **a** Mortality in children aged 0–4 from acute lower respiratory disease (ALRI). **b** Mortality in adults aged > 18 years old from chronic obstructive pulmonary disease (COPD). **c** Mortality in adults aged > 18 years old from ischaemic heart disease (IHD). **d** Mortality in adults aged > 18 years old from lung cancer (LC). **e** Mortality in adults aged > 18 years old from stroke and **f** post-neonatal all-cause infant mortality. The blue and wine circles represent the attributable proportion (%) and attributable cases per 100,000 people

Based on the PM_2.5_ estimation over the study period, long-term health effects were estimated, as presented in Fig. 4. A total of 24 ALRI deaths per 100,000 inhabitants in children under 4 years old were attributable to PM exposure each year in the northern Caribbean region of Colombia. This represents 11.6% of the total ALRI deaths in the study area (Supplementary Table S4). Similar proportions of cases attributable to air pollution have been found in larger populations (> 8 million). A quantitative risk assessment related to annual mortality in residents of Tehran (Iran) due to ALRI revealed 23 cases attributable to air pollution per 100,000 inhabitants (Ansari and Ehrampoush 2019).

An average of 11 annual COPD deaths were estimated to be associated with long-term exposure to PM_2.5_ estimation in the northern Caribbean region over the period 2011–2019. This represents an average of 17 cases per 100,000 inhabitants per year and accounts for 16.1% of COPD deaths annually. In cities with populations larger than the baseline of our study, similar values of 23.8 COPD mortality cases per 100,000 people have been found in Tehran during 2017–2018. According to Barzeghar et al. (2020), the annual percentage of COPD deaths related to prolonged exposure to particulate matter in Tabriz in 2006–2017 was 23.4%. Vehicular traffic and exposure to biomass smoke are considered environmental risk factors associated with COPD. It was reported that higher traffic density was associated with significantly lower forced expiratory volume in 1 s and forced vital capacity in women (Ko and Hui 2012). Furthermore, a study of hospital admissions related to heart and lung disease in 10 US cities showed that there was a 2.5% increase (95% CI: 1.8–3.3) in admissions for COPD per 10 µg m^−3^ increase in PM_10_ (Zanobetti et al. 2000).

The mean annual deaths of IHD due to prolonged PM_2.5_ exposure were 49 per 100,000 people over the period 2011–2019. This mean value is lower than those reported in other studies. Ansari and Ehrampoush (2019) reported that 112 deaths of IHD per 100,000 inhabitants were attributable to air pollution in Tehran (Iran) in 2017–2018. Hadei et al. (2017) conducted a study in 10 cities in Iran using AirQ+ and estimated that the average excess IHD mortality due to fine PM exposure across all cities was 84 per 100,000 inhabitants. The high mortality rate from IHD in the aforementioned studies may be related to the higher-risk populations.

Annual deaths from LC attributable to air pollution was 8 per 100,000 inhabitants. This signifies that an average of 9.1% of the deaths caused by LC in the region from 2011 to 2019 was due to prolonged PM_2.5_ exposure. Ansari and Ehrampoush (2019) estimated a death proportion attributable to LC of 17.8% in cities with 18 million inhabitants, almost double the value estimated in this study. Based on a case–control study in a population designed to investigate the genetics and environmental determinants of lung cancer in northwestern Italy, it was estimated that each 10 µg m^−3^ increase in coarse particles increased the cancer risk ratio by 1.28 (95% CI: 0.95–1.72) (Consonni et al. 2018). Long-term exposure to PM is associated with the development of lung cancer; the risk ratio in men was estimated to be 1.14–1.18, while that in women was estimated to be 1.04–1.13 (Lee et al. 2022). Ciabattini et al. (2021) conducted a systematic review and meta-analysis of studies on particulate matter exposure and lung cancer risk. They found that the RR for a 10 µg m^−3^ increase in fine PM_2.5_ was 1.16 (95% CI: 1.09–1.23), while the corresponding relative risk for PM_10_ exposure was 1.23 (95% CI: 1.05–1.40). It is possible that 148 cases of post-neonatal infant mortality (all-cause) were directly related to prolonged exposure to PM_10_ in the northern Caribbean region of Colombia from 2011 to 2019. This represents 5.4% of annual post-neonatal infant (1–12 months) deaths in the study area.

The proportion of stroke deaths attributable to prolonged exposure to PM_2.5_ in the study region for the period 2011–2019 was estimated to be 18.9%. Barzeghar et al. (2020) evaluated the effects of prolonged PM_2.5_ exposure in an Iranian city of approximately 1.5 million inhabitants. They found that monthly exposure to PM_10_ concentrations greater than 50 µg m^−3^ increased the risk of stroke by 26.7%. Several studies have found significant correlations between PM exposure and stroke occurrences. Shah et al. (2015) used a meta-analysis of observational studies in 28 countries to determine that larger particles exert local pulmonary effects while fine or ultrafine particles cause additional systemic cardiovascular effects. They found that PM exposure was associated with acute cardiovascular events mainly via the PM_2.5_ fraction. Prolonged exposure to fine PM negatively affects the activity of the sympathetic nervous system, leading to vasoconstriction, increased blood pressure, ischemia, and risk of thrombosis (Lucking et al. 2011). Increased rates of stroke deaths (33.9%, *P* < 0.05) were observed when PM_10_ concentrations were above 37 µg m^−3^ (Knezovic et al. 2018). However, a negative association was also found in another study, which suggested a 14.6% decrease in the risk of hemorrhagic stroke (95% CI 0.7% to 26.5%) per 10 µg m^−3^ increase in PM_10_ concentration. (Butland et al. 2017). Variations in air quality due to PM_2.5_ dust from the Sahara were estimated to contribute to 1% of annual long-term mortality cases in the region. These estimates were higher (up to 3%) during the years 2012, 2014, and 2015, when PM_2.5_ contributions significantly increased regional ambient PM concentrations.

The prevalence of bronchitis in children over 9 years of age due to long-term exposure to PM_10_ in the northern Caribbean region of Colombia is shown in Table 3. The highest percentages of AP (> 14%) were observed during the years with the highest mean annual PM_10_ concentrations in the region (> 35 µg m^−3^). In the study region, there were on average an estimated 114 cases of bronchitis in children per year. The average risk of cases attributable to PM_10_ exposure per 100,000 inhabitants was 109 per year. The average percentage of cases per year attributable to PM_10_ exposure over the period 2011–2019 was higher in the industrial zone (17%) than in the coastal zone (11%). Children represent the population subset that is most vulnerable to the impacts of air pollution (Kulkarni and Grigg 2008). Lower respiratory tract infections are a leading contributor to disease burden in low- and middle-income countries (WHO 2021b). Exposure to PM in early life causes impaired lung growth and increases the likelihood of developing the subsequent disease (Shao et al. 2020). Exposure to PM_10_ affects children during the fetal development as well as after birth (Mahapatra et al. 2020). The estimated AP for the prevalence of bronchitis in children due to exposure to PM_10_ was between 8 and 15% of the total cases in the region. In the coastal and industrial zones, the proportion of premature deaths (bronchitis in children) due to PM_10_ exposure was higher during 2014 at 14%. The percentage ratio of attributable deaths shows that the health impact of PM pollution was higher in the industrial zone (Table 4).

**Table 3:** Estimated attributable proportion (%) with 95% CI of all-cause mortality per year due to long-term exposure to PM_10_ greater than 15 μg m^−3^

	Northern Caribbean region	Coastal zone	Industrial zone	Mountainous zone
2011	11.70%	9.1% (3.8–19.9%)	16.3% (7.4–26.3%)	16.3% (5.1–22.5%)
2012	13.30%	11.1% (5.3–.22.9%)	19.5% (7.9–27.2%)	17.6% (5.5–23.3%)
2013	13.50%	11.5% (4.8–21.9%)	19.4% (8.6–28.2%)	17.1% (5.6–23.5%)
2014	14.30%	11.8% (5.6–25.0%)	20.0% (7.8–27.0%)	18.3% (7.5–26.6%)
2015	16.90%	14.3% (6.4–26.3%)	24.6% (8.6–28.2%)	20.9% (7.2–26.1%)
2016	16.20%	11.2% (4.7–23.3%)	20.9% (7.5–26.6%)	26.8% (11.2–32.3%)
2017	15.30%	12.3% (5.3–24.4%)	17.0% (7.7–26.9%)	21.1% (7.0–25.8%)
2018	15.20%	13.5% (6.2–25.8%)	16.5% (7.4–26.4%)	19.6% (7.0–25.8%)
2019	13.20%	10.5% (4.7–23.4%)	19.7% (7.2–26.1%)	18.0% (6.5–25.0%)

**Table 4:** Estimated attributable proportion (%) (number of excess cases) per year of the prevalence of bronchitis in children (aged 0–4) to exposure to PM_10_ in the study region between 2011 and 2019

	Northern Caribbean region	Coastal zone	Industrial zone	Mountainous zone
2011	8.0% (41)	5.0% (0–11.2%) (12)	15.7% (0–31.7%) (13)	8.6% (0–18.4%) (16)
2012	13.0% (80)	12.8% (0–20.1%) (47)	17.5% (0–34.4%) (18)	10.3% (0–21.2%) (15)
2013	12.9% (91)	11.6% (0–17.3%) (40)	19.2% (0–38.0%) (30)	10.4% (0–22.0%) (21)
2014	14.8% (136)	14.0% (0–22.3%) (77)	16.6% (0–33.9%) (26)	15.8% (0–32.5%) (33)
2015	15.0% (155)	12.9% (0–26.8%) (71)	19.1% (0–38.0%) (53)	14.9% (0–30.8%) (31)
2016	13.1% (100)	7.8% (0–16.8%) (32)	16.2% (0–32.6%) (41)	27.3% (0–51.7%) (27)
2017	11.5% (105)	9.6% (0–20.4%) (61)	16.5% (0–33.6%) (29)	14.9% (0–29.8%) (15)
2018	13.4% (122)	12.2% (0–25.3%) (70)	15.6% (0–31.8%) (37)	15.0% (0–29.9%) (15)
2019	9.6% (199)	7.9% (0–16.9%) (119)	15.1% (0–30.7%) (58)	12.8% (0–27.1%) (22)

The APs estimated for the incidence of chronic bronchitis in adults aged > 18 years old between 2011 and 2019 due to exposure to PM_10_ were less than 18%, 26.4%, and 37.1% in the coastal, industrial, and mountainous zones, respectively. The highest estimated APs occurred in the industrial zone (Table 5). The estimated total number of cases attributable to chronic bronchitis in adults in the northern Caribbean region of Colombia over the study period averaged 207 per year. This represents an average of 40 cases per 100,000 inhabitants per year. The number of cases per 100,000 inhabitants per year in the industrial zone was 2.34 and 1.40 times greater than the number of cases in the coastal and mountainous zones, respectively.

**Table 5:** Estimated attributable proportion (%) (number of excess cases) of the incidence of chronic bronchitis in adults (> 18 years old) to exposure to PM_10_ in the study region between 2011 and 2019

	Northern Caribbean region	Coastal zone	Industrial zone	Mountainous zone
2011	11.3% (90)	7.3% (2.6–11.1%) (33)	21.7% (8.4–31.2%) (33)	12.5% (4.6–18.8%) (24)
2012	14.7% (123)	13.3% (4.9–20.0%) (70)	23.1% (7.8–29.7%) (25)	13.9% (5.2–21.1%) (28)
2013	14.7% (139)	11.4% (4.2–17.2%) (68)	26.1% (10.1–37.9%) (46)	14.5% (5.5–21.9%) (25)
2014	17.7% (198)	14.8% (5.5–22.2%) (105)	23.3% (8.8–34.1%) (37)	22.3% (8.5–32.4%) (56)
2015	20.5% (244)	18.0% (6.8–26.7%) (111)	26.4% (10.8–36.4%) (61)	21.1% (8.0–30.7%) (72)
2016	17.6% (218)	11.0% (4.1–16.8%) (87)	22.3% (8.7–33.3%) (56)	37.1% (15.1–51.5%) (75)
2017	11.4% (165)	9.6% (0–20.4%) (94)	16.5% (6.4–26.6%) (40)	14.3% (0–29.8%) (31)
2018	18.3% (307)	17.0% (6.4–25.2%) (190)	21.6% (8.2–29.8%) (62)	20.1% (7.7–29.8%) (55)
2019	13.9% (383)	11.2% (4.1–16.9%) (206)	20.7% (7.8–32.4%) (96)	18.2% (6.9–27.0%) (81)

The annual mean PM concentrations based on the observational data were compared to the estimated values for the same station sites in each zone for the period 2011–2019. Statistical metrics of the model performance are displayed in Table S2 and Fig. S3. The estimated results gave a slight underestimation of PM_10_ concentrations in the coastal zone and industrial zone (biases of − 5.83 µg m^−3^ and − 9.10 µg m^−3^, respectively). On the other hand, estimated values showed good correspondence with observed values in the coastal zone (*r* = 0.63) and mountainous zone (*r* = 0.68). The NRMSE values were 0.43, 0.41, and 0.48 for the stations of the coastal, industrial, and mountainous zones, respectively. These values meet the acceptance criteria given by Kumar et al. (2006) for evaluating dispersion models. The model reproduced the observed PM_10_ concentrations in the study area in a long-term emission scenario with reasonable accuracy. A strong downwind concentration gradient was predicted in the industrial and mountainous zones. This gradient is explained by industrial emissions and meteorological factors which are influenced by the topography. The model did not consider regional-synoptic factors such as the arrival of dust from the Sahara Desert and biomass burning occurring outside of the study region. However, the CALPUFF model consistently reproduced long-term PM_10_ concentrations. The RR reflects the rate of health impacts due to a change in exposure to air pollutants. An RR of 1.55% was estimated for the total mortality from PM_10_ exposure, which is lower than that found in other research. In an Iranian metropolis, Miri et al. (2016) estimated an RR of total mortality associated with PM_10_ pollution of 1.61%. On the other hand, the authors estimated an AP of 4.24% for PM_10_. In another study conducted in an arid region of northwest China, the AP for total mortality attributable to PM_10_ was reported as 3%, while mortality due to cardiovascular incidents was estimated at 4% (Luo et al. 2020).

**Fig. 5:**
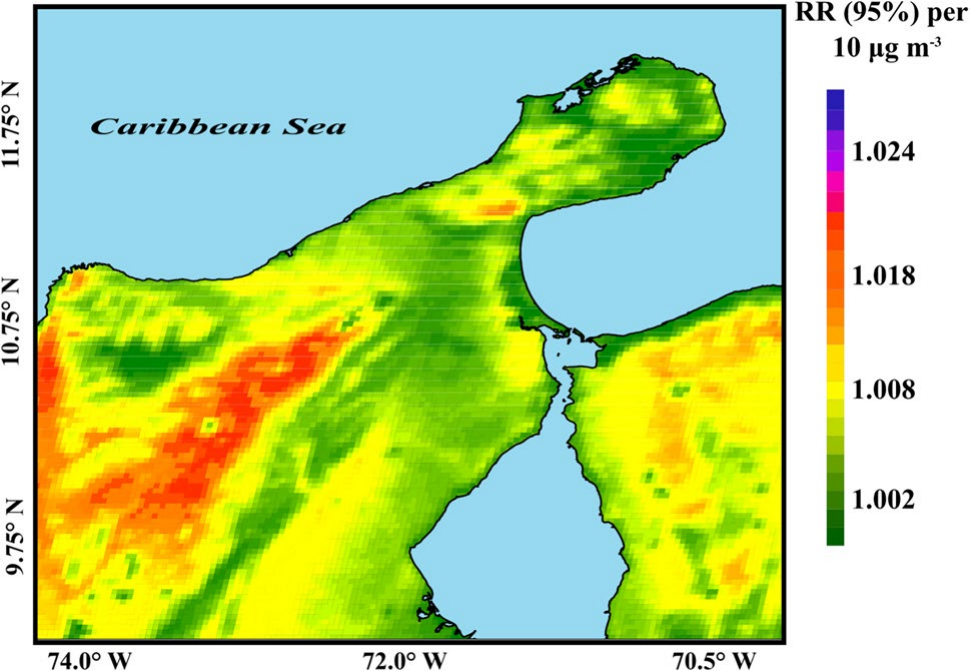
Relative risk of total mortality associated with PM_10_ exposure in the northern Caribbean region of Colombia

The RR of total mortality associated with long-term exposure to PM_10_ concentrations greater than 10 μg m^−3^ was 1.55% (1.015, 1.011–1.019) in the study region over the period 2011–2019. RR values varied spatially, as shown in Fig. 5. Mortality RR values of 1.04% (1.0104, 1.006–1.014), 1.95% (1.0195, 1.015–1.022), and 1.65% (1.0165, 1.012–1.021) were estimated for the coastal, industrial, and mountainous zones, respectively. These values were slight underestimates in the coastal and industrial zones due to the results produced by the dispersion model. The RR could increase significantly (up to 2.35%: 1.0235; 1.018–1.025) when Saharan dust contributions were temporally and spatially significant (as in the event presented in 2015). In Seoul (South Korea), RRs for PM_10_ mortality have been estimated to be 14.2% (1.1420–95% CI) and 2.3% (1.0230–95% CI) for post-neonatal children and people over 65 years of age, respectively (Ha et al. 2003). Research published before September 2021 uses a cutoff value of 10 µg m^−3^ for PM_2.5_ and 20 µg m^−3^ for PM_10_, based on the WHO 2005 Global Air Quality Guidelines (World Health Organization 2006; WHO 2016). In this study, we used cutoff values of 5 µg m^−3^ for PM_2.5_ and 15 µg m^−3^ for PM_10_, following the WHO 2021a, b, c air pollution guidelines (WHO 2021a). The new recommendations reflect recent evidence on the impact of lower concentrations of air pollution on human health and well-being, which are greater than previously considered. The RRs obtained in this study provide valuable information on the effects of air pollutants in the study area, allowing policy- and decision-makers to take appropriate actions to minimize the health effects of pollution.

### Limitations of this study

The main strength of our study is that our findings show that PM_10_ and PM_2.5_ exposure has a significant impact on mortality and morbidity indicators in the study region. Furthermore, a first approximation is made using RR values from meta-analysis of epidemiological studies and by selecting values according to the criteria of an air quality index, socioeconomic characteristics on human exposure, and transport. Although no epidemiological studies with study cohorts or time series have been conducted in the area, the RR values for each of the selected health endpoints fall within the range of values estimated from research conducted for similar geographic areas, populations, and PM levels. It is important to note that the RR values only consider the effects of individual pollutants and not the additive potential effects of multiple pollutants.

The use of aggregated data leads to limitations in this type of research. The calculations do not take into account multiple exposures or multiple contaminant scenarios. The estimates generated by AirQ+ carry some uncertainties, as they are based on information from concentration–response functions, which are based on several assumptions. The underlying scientific evidence on the health issues of ambient air pollution used in the AirQ+ software comes primarily from studies conducted in Western Europe and North America, which means that the applicability of the results generated for assessments conducted in Latin America is uncertain. In this study, the RR used in the AP calculations was derived from several studies that were based on different climatic and demographic characteristics. However, the use of AirQ+ is currently necessary for regional and national policymakers who must make decisions to prevent and control air pollution. Another significant limitation is related to the assumption of exposure of the target population; the concentrations assigned to zones were the result of averaging the concentrations of the PM_10_ stations on the surface of each zone, thus assuming no variabilities in exposures within zones. The absence of PM_2.5_ records from the years 2011–2017 is another factor that increases uncertainty. The PM_2.5_/PM_10_ conversion factor for estimating PM_2.5_ levels also increases uncertainty among the estimates for each disease in the PM-exposed population. Despite the limitations of the approach used herein and in other similar studies, the AirQ+ model is a useful tool that can assist in the formulation of public policy and in decision-making aimed at setting environmental and health standards to reduce the effects of air pollution on the population.

## Conclusion

The quantifiable issues of mortality and morbidity resulting from PM exposure between 2011 and 2019 in the northern Caribbean region of Colombia were investigated. This study used the AirQ+ model to estimate the APs of morbidity and mortality at the population level due to PM exposure. The main conclusions of this study are as follows:

PM concentrations in the industrial zone were higher than the levels recorded in the coastal and mountainous zones. the spatiotemporal variations were mainly attributable to coal mining activities. The highest annual PM_2.5_ concentrations in the region were recorded during 2014 and 2015, mainly due to dust particles arriving from the Sahara Desert.

Based on our estimates, the number of yearly deaths per 100,000 inhabitants attributable to air pollution via ALRI (in children 0–5 years of age), COPD, IHD, LC, stroke, and post-neonatal infant mortality (in children 0–12 months) were 33, 17, 49, 8, 27, and 345, respectively. The evidence from this study suggests that long-term planning is needed to actively reduce PM pollution in the northern Caribbean region of Colombia, particularly in areas wherein industrial activities take place.

## Supplementary information

Below is the link to the electronic supplementary material.

Supplementary file1 (DOCX 457 KB)

## Data Availability

The datasets used in the current study are available from the corresponding author on reasonable request.

## Nomenclature

PM: Particulate matter
ALRI: Acute lower respiratory disease
COPD: Chronic obstructive pulmonary disease
IHD: Ischemic heart disease
LC: Lung cancer
RR: Relative risk
AP: Attributable proportion

## Code availability

The code used in the current study is available from the corresponding author on reasonable request.

## References

[CR1] Ansari M, Ehrampoush MH (2019). Meteorological correlates and AirQ+ health risk assessment of ambient fine particulate matter in Tehran. Iran Environ Res.

[CR2] Arregocés HA, Rojano R, Angulo L, Restrepo G (2018). Intake fraction of PM10 from coal mine emissions in the North of Colombia. J Environ Public Health.

[CR3] Barzeghar V, Sarbakhsh P, Hassanvand MS (2020). Long-term trend of ambient air PM10, PM2. 5, and O3 and their health effects in Tabriz city, Iran, during 2006–2017. Sustain Cities Soc.

[CR4] Bayona Velásquez EM (2016) Coal production and economic growth in the Caribbean mining region in Colombia. Rev Econ del Caribe 17:1–38. 10.14482/ecoca.17.8452

[CR5] Bozlaker A, Prospero JM, Fraser MP, Chellam S (2013). Quantifying the contribution of long-range Saharan dust transport on particulate matter concentrations in Houston, Texas, using detailed elemental analysis. Environ Sci Technol.

[CR6] Brito J, Bernardo A, Gonçalves LL (2022). Atmospheric pollution and mortality in Portugal: quantitative assessment of the environmental burden of disease using the AirQ+ model. Sci Total Environ.

[CR7] Burnett R, Chen H, Szyszkowicz M (2018). Global estimates of mortality associated with long-term exposure to outdoor fine particulate matter. Proc Natl Acad Sci.

[CR8] Butland BK, Atkinson RW, Crichton S (2017). Air pollution and the incidence of ischaemic and haemorrhagic stroke in the South London Stroke Register: a case–cross-over analysis. J Epidemiol Community Heal.

[CR9] Cao J, Yang C, Li J (2011). Association between long-term exposure to outdoor air pollution and mortality in China: a cohort study. J Hazard Mater.

[CR10] Carungo M, Dentali F, Mathieu G, Fontanella A, Mariani J, Bordini L (2018). PM10 exposure is associated with increased hospitalizations for respiratory syncytial virus bronchiolitis among infants in Lombardy. Italy Environ Res.

[CR11] Chen J, Hoek G (2020). Long-term exposure to PM and all-cause and cause-specific mortality: a systematic review and meta-analysis. Environ Int.

[CR12] Chin M, Ginoux P, Kinne S (2002). Tropospheric aerosol optical thickness from the GOCART model and comparisons with satellite and Sun photometer measurements. J Atmos Sci.

[CR13] Choi JH, Kim JS, Kim YC (2004). Comparative study of PM2. 5-and PM10-induced oxidative stress in rat lung epithelial cells. J Vet Sci.

[CR14] Ciabattini M, Rizzello E, Lucaroni F (2021). Systematic review and meta-analysis of recent high-quality studies on exposure to particulate matter and risk of lung cancer. Environ Res.

[CR15] Consonni D, Carugno M, De Matteis S (2018). Outdoor particulate matter (PM10) exposure and lung cancer risk in the EAGLE study. PLoS ONE.

[CR16] Ćurić M, Zafirovski O, Spiridonov V (2022) Air quality and health. In: Essentials of medical meteorology. Springer, Cham, pp 143–182. 10.1007/978-3-030-80975-1_8

[CR17] DANE (2018a) Censo de Colombia de 2018a ( updated 14 July 2021). In: Estadísticas por tema. https://www.dane.gov.co/index.php/estadisticas-por-tema#estadisticas-por-tema. Accessed 14 Jul 2021

[CR18] DANE (2018b) Censo de Colombia de 2018b ( updated 09 October 2021). https://www.dane.gov.co/index.php/estadisticas-por-tema/demografia-y-poblacion/proyecciones-de-poblacion. Accessed 14 Jul 2021

[CR19] Doria-Argumedo C, Fagundo-Castillo J (2017). Metal levels in atmospheric particles in the coal mining zone, northern Colombia. Iteckne.

[CR20] Eastwood P (2008) Particulate emissions from vehicles. John Wiley & Sons, Chichester, West Sussex, UK

[CR21] Echeverri M, Anderson D, Haas JM (2020). Testing the preliminary validity of a multidimensional framework for studying the effects of cancer health literacy on cancer screening behaviors among diverse populations. Int J Environ Res Public Health.

[CR22] Enders C, Pearson D, Harley K, Ebisu K (2019). Exposure to coarse particulate matter during gestation and term low birthweight in California: variation in exposure and risk across region and socioeconomic subgroup. Sci Total Environ.

[CR23] Espitia-Pérez L, da Silva J, Espitia-Pérez P (2018). Cytogenetic instability in populations with residential proximity to open-pit coal mine in Northern Colombia in relation to PM10 and PM2.5 levels. Ecotoxicol Environ Saf.

[CR24] Götschi T, Heinrich J, Sunyer J, Künzli N (2008). Long-term effects of ambient air pollution on lung function: a review. Epidemiology.

[CR25] GMAO (2015) MERRA-2 tavgU_2d_lnd_Nx: 2d, diurnal, time-averaged, single-level, assimilation, aerosol Diagnostics V5.12.4. Greenbelt, MD: Goddard Earth Sciences Data and Information Services Center (GES DISC). 10.5067/KPUMVXFEQLA1

[CR26] Guzmán P, Tarín-Carrasco P, Morales-Suárez-Varela M, Jiménez-Guerrero P (2022). Effects of air pollution on dementia over Europe for present and future climate change scenarios. Environ Res.

[CR27] Ha E-H, Lee J-T, Kim H (2003). Infant susceptibility of mortality to air pollution in Seoul, South Korea. Pediatrics.

[CR28] Hadei M, Nazari SSH, Eslami A (2017). Distribution and number of ischemic heart disease (IHD) and stroke deaths due to chronic exposure to PM2. 5 in 10 cities of Iran (2013–2015); an AirQ+ modelling. J Air Pollut Heal.

[CR29] Han C, Lim Y, Jung K, Hong Y (2017). Association between ambient particulate matter and disorders of vestibular function. Environ Res.

[CR30] Hansell A, Ghosh RE, Blangiardo M (2016). Historic air pollution exposure and long-term mortality risks in England and Wales: prospective longitudinal cohort study. Thorax.

[CR31] Hassanvand MS, Naddafi K, Kashani H, Faridi S, Kunzli N, Nabizadeh R, Momeniha F, Gholampour A, Arhami M, Zare A (2017). Short-term effects of particle size fractions on circulating biomarkers of inflammation in a panel of elderly subjects and healthy young adults. Environ Pollut.

[CR32] Health Effects Institute (2019) State of global air 2019. Special Report

[CR33] Hystad P, Larkin A, Rangarajan S (2020). Associations of outdoor fine particulate air pollution and cardiovascular disease in 157 436 individuals from 21 high-income, middle-income, and low-income countries (PURE): a prospective cohort study. Lancet Planet Heal.

[CR34] INS (2018) Instituto Nacional de Salud. Carga de enfermedad ambiental en Colombia. Inf Técnico Espec 10. https://www.ins.gov.co/Paginas/Inicio.aspx

[CR35] INS (2021) Instituto Nacional de Salud. Estadísticas de Vigilancia Rutinaria. http://portalsivigila.ins.gov.co/Paginas/Vigilancia-Rutinaria.aspx

[CR36] Kang J-H, Keller JJ, Chen C-S, Lin H-C (2012). Asian dust storm events are associated with an acute increase in pneumonia hospitalization. Ann Epidemiol.

[CR37] Kim H, Kim J, Kim S (2017). Cardiovascular effects of long-term exposure to air pollution: a population-based study with 900 845 person-years of follow-up. J Am Heart Assoc.

[CR38] Knezovic M, Pintaric S, Jelavic MM (2018). The role of weather conditions and normal level of air pollution in appearance of stroke in the region of Southeast Europe. Acta Neurol Belg.

[CR39] Ko FWS, Hui DSC (2012). Air pollution and chronic obstructive pulmonary disease. Respirology.

[CR40] Koengkan M, Fuinhas JA, Silva N (2021). Exploring the capacity of renewable energy consumption to reduce outdoor air pollution death rate in Latin America and the Caribbean region. Environ Sci Pollut Res.

[CR41] Kulkarni N, Grigg J (2008). Effect of air pollution on children. Paediatr Child Health (Oxford).

[CR42] Kumar A, Dixit S, Varadarajan C (2006). Evaluation of the AERMOD dispersion model as a function of atmospheric stability for an urban area. Environ Prog.

[CR43] Kutralam-Muniasamy G, Pérez-Guevara F, Martínez IE, Chari SV (2021). Particulate matter concentrations and their association with COVID-19-related mortality in Mexico during June 2020 Saharan dust event. Environ Sci Poll Res.

[CR44] Lee HW, Kang S-C, Kim S-Y, Cho Y-J, Hwang S-S (2022). Long-term exposure to PM10 increases lung cancer risks: a cohort analysis. Cancer Res Treat.

[CR45] Lindstrom MJ, Bates DM (1990). Nonlinear mixed effects models for repeated measures data. Biometrics.

[CR46] Lucking AJ, Lundbäck M, Barath SL (2011). Particle traps prevent adverse vascular and prothrombotic effects of diesel engine exhaust inhalation in men. Circulation.

[CR47] Luo H, Guan Q, Lin J (2020). Air pollution characteristics and human health risks in key cities of northwest China. J Environ Manage.

[CR48] Mahapatra B, Walia M, Avis WR, Saggurti N (2020). Effect of exposure to PM10 on child health: evidence based on a large-scale survey from 184 cities in India. BMJ Glob Heal.

[CR49] Mahato S, Pal S, Ghosh KG (2020). Effect of lockdown amid COVID-19 pandemic on air quality of the megacity Delhi.

[CR50] Malhotra J, Malvezzi M, Negri E (2016). Risk factors for lung cancer worldwide. Eur Respir J.

[CR51] Martin R, Dowling K, Pearce D (2014). Health effects associated with inhalation of airborne arsenic arising from mining operations. Geosciences.

[CR52] Méndez JF, Pinto-Herrera LC, Belalcázar-Cerón LC (2018). Study of a Saharan Dust Intrusion into the Colombian Atmosphere. Rev Ing Univ Medellín.

[CR53] Middleton N, Yiallouros P, Kleanthous S (2008). A 10-year time-series analysis of respiratory and cardiovascular morbidity in Nicosia, Cyprus: the effect of short-term changes in air pollution and dust storms. Environ Heal.

[CR54] MinSalud (2021) Minsalud comprometido con la calidad del aire. https://www.minsalud.gov.co/Paginas/Minsalud-comprometido-con-la-calidad-del-aire-.aspx

[CR55] Miri M, Derakhshan Z, Allahabadi A (2016). Mortality and morbidity due to exposure to outdoor air pollution in Mashhad metropolis, Iran. The AirQ model approach. Environ Res.

[CR56] Mirzaei A, Tahriri H, Khorsandi B (2021). Comparison between AirQ+ and BenMAP-CE in estimating the health benefits of PM 2.5 reduction. Air Qual Atmos Heal.

[CR57] Momtazan M, Geravandi S, Rastegarimehr B, Valipour A, Ranjbarzadeh A, Yari AR (2018). An investigation of particulate matter and relevant cardiovascular risks in Abadan and Khorramshahr in 2014–2016. Toxin Rev.

[CR58] Mudu P, Gapp C, Dunbar M (2018) AirQ+: example of calculations. No. WHO/EURO: 2016-4103-43862-61760. World Health Organization. Regional Office for Europe, 2016

[CR59] Neophytou AM, Yiallouros P, Coull BA (2013). Particulate matter concentrations during desert dust outbreaks and daily mortality in Nicosia, Cyprus. J Expo Sci Environ Epidemiol.

[CR60] Osornio-Vargas ÁR, Bonner JC, Alfaro-Moreno E (2003). Proinflammatory and cytotoxic effects of Mexico City air pollution particulate matter in vitro are dependent on particle size and composition. Environ Health Perspect.

[CR61] Ouidir M, Lepeule J, Siroux V, Malherbe L, Meleux F, Rivière E, Launay L, Zaros C, Cheminat M, Charles M-A, et al. (2017) Is atmospheric pollution exposure during pregnancy associated with individual and contex-tual characteristics? A nationwide study in France. J Epidemiol Community Health 71(10):1026–1036. https://www.jstor.org/stable/26383982 10.1136/jech-2016-208674 28830952

[CR62] Priestley MB (1981) Spectral analysis and time series. Academic Press, San Diego

[CR63] Randles CA, Da Silva AM, Buchard V (2017). The MERRA-2 aerosol reanalysis, 1980 onward. Part I: System description and data assimilation evaluation. J Clim.

[CR64] Rojano R, Angulo L, Restrepo G (2013). Niveles de Partículas Suspendidas Totales (PST), PM10 y PM2.5 y su Relación en Lugares Públicos de la Ciudad Riohacha, Caribe Colombiano. (Spanish). Levels Total suspended Part (TSP), PM10 PM25 their Relatsh public places city Riohacha. Colomb Caribbean.

[CR65] Rovira J, Domingo JL, Schuhmacher M (2020). Air quality, health impacts and burden of disease due to air pollution (PM10, PM2. 5, NO2 and O3): Application of AirQ+ model to the Camp de Tarragona County (Catalonia, Spain). Sci Total Environ.

[CR66] Setti L (2020) SARS-Cov-2 RNA found on particulate matter of Bergamo in Northern Italy. SIMA propose to use it worldwide as “indicator” of COVID-19 relapses. https://www.iscleanair.com/wp/wp-content/uploads/2020/04/PRESS-RELEASE-covid-pm_24.04.20.pdf

[CR67] Setti L, Passarini F, De Gennaro G, Barbieri P, Perrone MG, Borelli M, Palmisani J, Di Gilio A, Torboli V, Fontana F, Clemente L, Pallavicini A, Ruscio M, Piscitelli P, Miani A (2020). SARS-Cov-2RNA found on particulate matter of Bergamo in Northern Italy: first evidence. Environ Res.

[CR68] Shah ASV, Lee KK, McAllister DA, Hunter A, Nair H, Whiteley W, Mills NL (2015). Short term exposure to air pollution and stroke: systematic review and meta-analysis. Bmj-British Medical Journal.

[CR69] Shao J, Zosky GR, Wheeler AJ (2020). Exposure to air pollution during the first 1000 days of life and subsequent health service and medication usage in children. Environ Pollut.

[CR70] Torres P, Ferreira J, Monteiro A, Costa S, Pereira MC, Madureira J, Mendes A, Teixeira JP (2018). Air pollution: a public health approach for Portugal. Sci Total Environ.

[CR71] UNIDO (2019) Health and pollution action plan. In: Mitigating Toxic Heal. Expo. Lowand Middle-Income Ctries. /www.unido.org/sites/default/files/files/2019-10/Colombia HPAP.English.pdf

[CR72] WHO (2018) Air pollution. In: World Heal. Organ. http://www.who.int/airpollution/en/. Accessed 20 Jul 2018

[CR73] WHO (2021a) New WHO Global Air Quality Guidelines aim to save millions of lives from air pollution. https://www.who.int/news/item/22-09-2021a-new-who-global-air-quality-guidelines-aim-to-save-millions-of-lives-from-air-pollution

[CR74] WHO (2020) Health impact assessment of air pollution: AirQ+ life table manual (No. WHO/EURO: 2020-1559-41310-56212). World Health Organization. Regional Office for Europe. https://apps.who.int/iris/handle/10665/337683

[CR75] WHO (2021b) WHO global air quality guidelines: particulate matter (PM2.5 and PM10), ozone, nitrogen dioxide, sulfur dioxide and carbon monoxide. https://www.who.int/publications/i/item/9789240034228 34662007

[CR76] WHO (2016) WHO expert consultation: available evidence for the future update of the WHO Global Air Quality Guidelines (AQGs): WHO/EURO: 2016-4105-43864-61762. World Health Organization. Regional Office for Europe. Geneva, Switzerland. https://apps.who.int/iris/handle/10665/341714

[CR77] WHO (2006) WHO Air quality guidelines for particulate matter, ozone, nitrogen dioxide and sulfur dioxide: Global update 2005: Summary of risk assessment (No. WHO/SDE/PHE/OEH/06.02). Geneva: World Health Organization. https://apps.who.int/iris/handle/10665/69477 34662007

[CR78] Worldometers (2020) Coal Production by Country. https://www.worldometers.info/coal/coal-production-by-country/. Accessed 4 Apr 2021

[CR79] Wu X, Nethery R, Benjamin M, Braun D, Dominici F (2020). Exposure to air pollution and COVID-19 mortality in the United States: a nationwide cross-sectional study. MedRxiv.

[CR80] Xu G, Jiao L, Zhang B (2017). Spatial and temporal variability of the PM2. 5/PM10 ratio in Wuhan, Central China. Aerosol Air Qual Res.

[CR81] Yang J, Zhou M, Li M (2020). Fine particulate matter constituents and cause-specific mortality in China: a nationwide modelling study. Environ Int.

[CR82] Zanobetti A, Schwartz J, Dockery DW (2000). Airborne particles are a risk factor for hospital admissions for heart and lung disease. Environ Health Perspect.

