# Supplementary material for: Health risk assessment for particulate matter: application of AirQ+ model in the northern Caribbean region of Colombia
Source: Air Qual Atmos Health. 2023 Feb 15;16(5):897–912. doi: 10.1007/s11869-023-01304-5 (PMC9930048; doi:10.1007/s11869-023-01304-5)
Supplement: Supplementary file 1 — Supplementary file1 (DOCX 457 KB) [file 11869_2023_1304_MOESM1_ESM.docx]

**Supplementary Information**

| 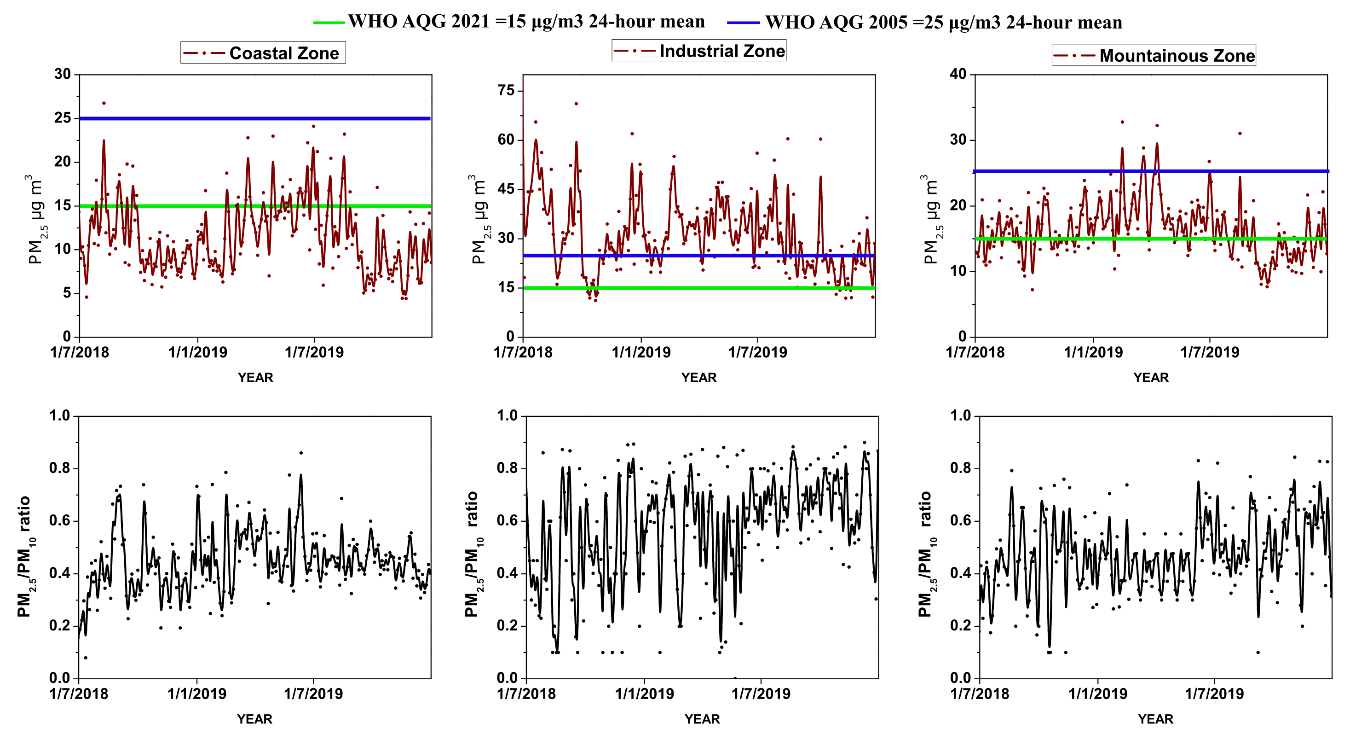 |
| --- |
| Figure S1. Daily PM_2.5_ concentrations in the northern Caribbean region of Colombia from July 2018 to December 2019 |

| 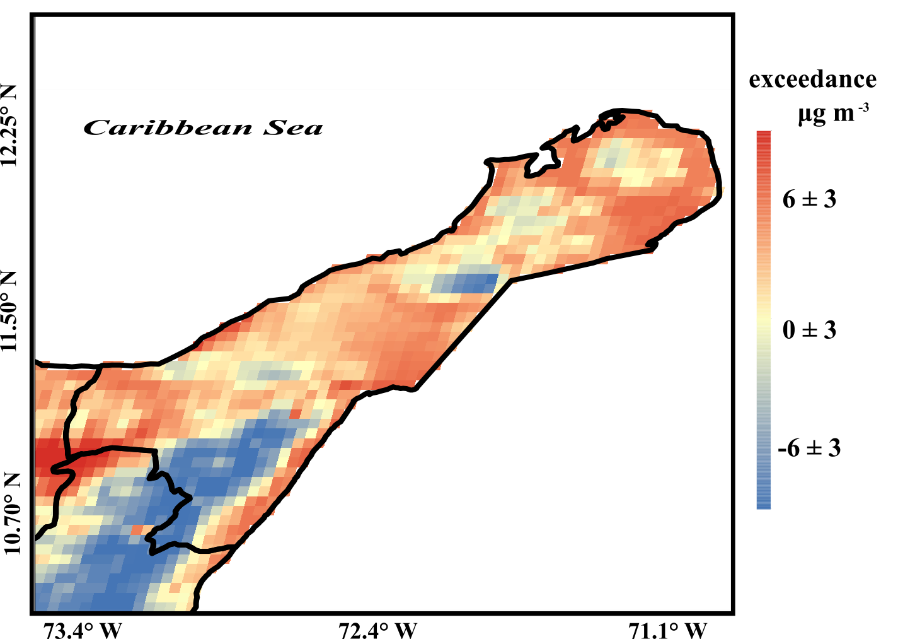 |
| --- |
| Figure S2. Representative zones (yellow areas) and zones with excess average PM_10_ concentration according to surface monitoring station records during the period 2011–2019. Surface station records vs. dispersion modeling estimates integrating the population density of each zone. The spatial distribution of PM_10_ was obtained through spatial interpolation performed using the deterministic inverse distance weighting (IDW) method for the surface stations located in the study domain. |

Table S1. Configuration of the WRF model for simulations in the northern Caribbean region of Colombia

| **Dynamics** | **Non-hydrostatic** |
| --- | --- |
| Observation period | January 2011 to December 2019 |
| Resolution | Domain 1: 36 × 36 km, Domain 2: 12 × 12 km, Domain 3: 4 × 4 km |
| Area covered | 7.43 to 15.54°N, -76.92 to -68.63°E |
| Projection | Mercator |
| Horizontal grid system | Arakawa-C grid |
| Vertical resolution | 38 vertical levels |
| spin-up time | 15 days |
| Microphysics scheme | Thompson scheme |
| Longwave and shortwave radiation Scheme | RRTMG |
| PBL schemes | MYNN |
| Cumulus parameterization | New Tiedtke scheme |

Table S2. Summary of the results of CALPUFF-CALMET-WRF evaluation for PM_10_ concentrations for 2011–2019 in the northern Caribbean region of Colombia

| **Statistics** | **Unit** | **Range** | **Best value** | **Performance of the CALPUFF mode** | | |
| --- | --- | --- | --- | --- | --- | --- |
|  |  |  |  | **Coastal Zone** | **Industrial Zone** | **Mountainous Zone** |
| $BIAS=\sum_{t=1}^{n} \frac{\emptyset_{P}-\emptyset_{O}}{\emptyset_{O}}$ | µg m^-3^ | -∞ to ∞ | 0 | -5.83 | -9.1 | 3.21 |
| $RMSE=\sqrt{\sum_{t=1}^{n} \frac{{{(\emptyset}_{P}-\emptyset_{O})}^{2}}{n}}$ | µg m^-3^ | 0 to ∞ | 0 | 13.43 | 16.65 | 16.62 |
| $NRMSE=\frac{\sqrt{\sum_{t=1}^{n} \frac{{{(\emptyset}_{P}-\emptyset_{O})}^{2}}{n}}}{\bar{\emptyset_{O}}}$ | None | 0 to ∞ | 0 | 0.43 | 0.41 | 0.48 |
| $r=\frac{1}{(n-1)}\sum_{t=1}^{n} \left( \frac{\emptyset_{P}-\bar{\emptyset_{P}}}{\sigma_{P}} \right)\left( \frac{\emptyset_{O}-\bar{\emptyset_{O}}}{\sigma_{O}} \right)$ | None | -1 to 1 | 1 | 0.63 | 0.51 | 0.68 |
| $MG=e^{(\bar{\ln} C_{0}-\bar{\ln C_{p}})}$ | None | 0 to ∞ | 1 | 1.23 | 1.29 | 0.92 |

| 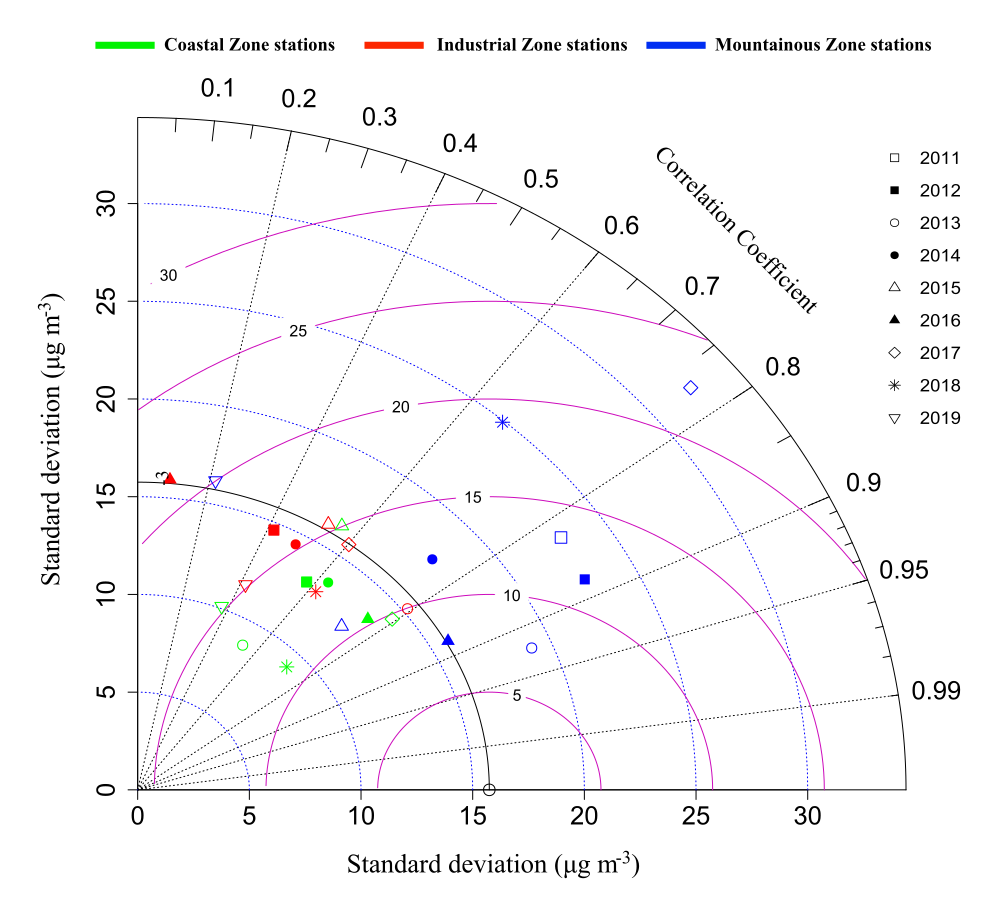 |
| --- |
| Figure S3. Taylor plot of annual PM_10_ concentrations vs. PM_10_ concentrations estimated using the CALPUFF-CALMET-WRF modeling system. |

Table S3. Monthly PM_10_ concentrations (µg m^-3^) in the Caribbean region of Colombia over the period 2011-2019.

|  | **Coastal Zone**  **(µg m^-3^)** | **Industrial Zone**  **(µg m^-3^)** | **Mountainous Zone**  **(µg m^-3^)** |
| --- | --- | --- | --- |
| JAN | 22 ± 8 | 35 ± 8 | 32 ± 12 |
| FEB | 27 ± 11 | 42 ± 11 | 39 ± 14 |
| MAR | 26 ± 10 | 46 ± 13 | 44 ± 15 |
| APR | 31 ± 15 | 50 ± 29 | 42 ± 18 |
| MAY | 37 ± 17 | 40 ± 13 | 36 ± 13 |
| JUN | 43 ± 26 | 42 ± 17 | 39 ± 18 |
| JUL | 35 ± 15 | 42 ± 12 | 37 ± 15 |
| AUG | 29 ± 10 | 37 ± 10 | 31 ± 11 |
| SEP | 26 ± 13 | 32 ± 10 | 28 ± 12 |
| OCT | 18 ± 10 | 28 ± 7 | 22 ± 7 |
| NOV | 16 ± 6 | 27 ± 8 | 19 ± 7 |
| DEC | 21 ± 7 | 30 ± 7 | 26 ± 11 |

Table S4. Estimated percentages of ALRI, COPD, IHD, LC, stroke, and post-neonatal infant mortality deaths attributable to PM_10_ exposure in the Northern Caribbean region of Colombia (2011-2019).

| **Year** | **Mortality** | **Northern Caribbean region** | **Number of Attributable Cases per 100,000 Population at Risk** |
| --- | --- | --- | --- |
| 2011 | ALRI (children 0-4) | 8% | 24 |
|  | COPD (adults 18+) | 12% | 19 |
|  | IHD (adults 18+) | 24% | 38 |
|  | LC (adults 18+) | 10% | 5 |
|  | Stroke (adults 18+) | 17% | 17 |
|  | Postneonatal infant mortality, allcause | 3% | 326 |
| 2012 | ALRI (children 0-4) | 9% | 11 |
|  | COPD (adults 18+) | 14% | 9 |
|  | IHD (adults 18+) | 26% | 35 |
|  | LC (adults 18+) | 9% | 6 |
|  | Stroke (adults 18+) | 19% | 21 |
|  | Postneonatal infant mortality, allcause | 5% | 321 |
| 2013 | ALRI (children 0-4) | 14% | 37 |
|  | COPD (adults 18+) | 17% | 8 |
|  | IHD (adults 18+) | 26% | 36 |
|  | LC (adults 18+) | 9% | 6 |
|  | Stroke (adults 18+) | 18% | 22 |
|  | Postneonatal infant mortality, allcause | 5% | 315 |
| 2014 | ALRI (children 0-4) | 9% | 22 |
|  | COPD (adults 18+) | 17% | 17 |
|  | IHD (adults 18+) | 28% | 33 |
|  | LC (adults 18+) | 6% | 8 |
|  | Stroke (adults 18+) | 19% | 22 |
|  | Postneonatal infant mortality, allcause | 6% | 343 |
| 2015 | ALRI (children 0-4) | 14% | 22 |
|  | COPD (adults 18+) | 19% | 16 |
|  | IHD (adults 18+) | 28% | 49 |
|  | LC (adults 18+) | 7% | 7 |
|  | Stroke (adults 18+) | 20% | 28 |
|  | Postneonatal infant mortality, allcause | 7% | 312 |
| 2016 | ALRI (children 0-4) | 14% | 35 |
|  | COPD (adults 18+) | 18% | 17 |
|  | IHD (adults 18+) | 29% | 58 |
|  | LC (adults 18+) | 14% | 6 |
|  | Stroke (adults 18+) | 20% | 25 |
|  | Postneonatal infant mortality, allcause | 5% | 387 |
| 2017 | ALRI (children 0-4) | 12% | 33 |
|  | COPD (adults 18+) | 16% | 19 |
|  | IHD (adults 18+) | 27% | 57 |
|  | LC (adults 18+) | 9% | 8 |
|  | Stroke (adults 18+) | 19% | 33 |
|  | Postneonatal infant mortality, allcause | 6% | 372 |
| 2018 | ALRI (children 0-4) | 13% | 56 |
|  | COPD (adults 18+) | 17% | 20 |
|  | IHD (adults 18+) | 27% | 61 |
|  | LC (adults 18+) | 10% | 12 |
|  | Stroke (adults 18+) | 19% | 37 |
|  | Postneonatal infant mortality, allcause | 7% | 519 |
| 2019 | ALRI (children 0-4) | 12% | 58 |
|  | COPD (adults 18+) | 16% | 26 |
|  | IHD (adults 18+) | 26% | 72 |
|  | LC (adults 18+) | 8% | 13 |
|  | Stroke (adults 18+) | 18% | 40 |
|  | Postneonatal infant mortality, allcause | 5% | 666 |
